# The relationship between dopamine receptor blockade and cognitive performance in schizophrenia: a [^11^C]-raclopride PET study with aripiprazole

**DOI:** 10.1038/s41398-018-0134-6

**Published:** 2018-04-24

**Authors:** Sangho Shin, Seoyoung Kim, Seongho Seo, Jae Sung Lee, Oliver D. Howes, Euitae Kim, Jun Soo Kwon

**Affiliations:** 1 Department of Neuropsychiatry, Seoul National University Bundang Hospital, Gyeonggi-do, 13620 Republic of Korea; 2 Department of Brain and Cognitive Sciences, College of Natural Science, Seoul National University, Seoul, 08826 Republic of Korea; 3 Department of Nuclear Medicine, Seoul National University College of Medicine, Seoul, 03080 Republic of Korea; 4 Institute of Psychiatry, Psychology and Neuroscience, King’s College London, London, SE5 8AF UK; 5 Medical Research Council Clinical Sciences Centre, London, W12 0NN UK; 6 Imperial College London, Hammersmith Hospital Campus, London, W12 0NN UK; 7 Department of Psychiatry, Seoul National University College of Medicine, Seoul, 03080 Republic of Korea; 8 Department of Neuropsychiatry, Seoul National University Hospital, Seoul, 03080 Republic of Korea; 9 Institute of Human Behavioral Medicine, SNU-MRC, Seoul, 03080 Republic of Korea

## Abstract

Aripiprazole’s effects on cognitive function in patients with schizophrenia are unclear because of the difficulty in disentangling specific effects on cognitive function from secondary effects due to the improvement in other schizophrenic symptoms. One approach to address this is to use an intermediate biomarker to investigate the relationship between the drug’s effect on the brain and change in cognitive function. This study aims to investigate aripiprazole’s effect on working memory by determining the correlation between dopamine D2/3 (D2/3) receptor occupancy and working memory of patients with schizophrenia. Seven patients with schizophrenia participated in the study. Serial positron emission tomography (PET) scans with [^11^C]raclopride were conducted at 2, 26, and 74 h after the administration of aripiprazole. The subjects performed the *N*-back task just after finishing the [^11^C]raclopride PET scan. The mean (±SD) D2/3 receptor occupancies were 66.9 ± 6.7% at 2 h, 65.0 ± 8.6% at 26, and 57.7 ± 11.2% at 74 h after administering aripiprazole. Compared with performance on the zero-back condition, performance in memory-loaded conditions (one-, two-, and three-back conditions) was significantly related to D2/3 receptor occupancy by aripiprazole (error rate: *ß* = −2.236, *t* = −6.631, df = 53.947, and *p* = 0.001; reaction time: *ß* = −9.567, *t* = −2.808, df = 29.967, and *p* = 0.009). Although the sample size was relatively small, these results suggest that aripiprazole as a dopamine-partial agonist could improve cognitive function in patients with schizophrenia.

## Introduction

Aripiprazole is a dopamine-partial agonist. This pharmacological profile has been proposed to offer efficacy against the cognitive and negative symptoms of schizophrenia, as well as positive symptoms^1^. Indeed, it has been reported that aripiprazole is safe and effective in improving cognitive and negative symptoms, as well as positive symptoms of schizophrenia^2–4^. However, there are other studies indicating that it may also cause cognitive dysfunctions in the aspects of verbal fluency and executive function in patients with schizophrenia^5^. The inconsistency may be due to variation in drug occupancy of dopamine receptors in the brain^6^.

In a previous study, we reported that striatal dopamine D2/3 (D2/3) receptor occupancies by aripiprazole were related to working memory in healthy volunteers^6^. We focused on the primary effects of aripiprazole on cognitive function and controlled the baseline performance by serial measurements of the relationship in the healthy volunteers. We found that greater striatal D2/3 receptor occupancy by aripiprazole was related to decreased performance in working-memory tasks. The result is consistent with previous studies conducted in healthy volunteers administered with haloperidol^7,8^ and a high dose of amisulpride^8^, demonstrating a decrease in cognitive performances, including attention, response time, and information processing associated with greater D2/3 receptor occupancy.

As mentioned above, aripiprazole acts as a potent dopamine-partial agonist. The partial agonist acts as an antagonist at D2 receptor in the state of excessive dopaminergic neurotransmission while behaving as an agonist in the state of low dopaminergic neurotransmission^9,10^. As the dopaminergic system in patients with schizophrenia is dysfunctional^11^, the effects of aripiprazole on cognitive function in patients with schizophrenia are likely to differ from those seen in healthy volunteers with a normal dopaminergic system.

This study aimed to determine the relationship between dopamine receptor occupancy by aripiprazole and change in working memory in patients with schizophrenia. As mentioned above, the change in psychotic symptoms and baseline performance of subjects could confound the effect of aripiprazole on cognitive function. Thus, we sought to determine the relationship in clinically stable patients with schizophrenia and obtained serial D2/3 receptor occupancies by using [^11^C]raclopride positron emission tomography (PET) and working-memory performances for 74 h after the last administration of aripiprazole to take the individual variation in cognitive performance into consideration.

## Materials and methods

The present study was approved by the Institutional Review Board of Seoul National University Hospital, Seoul, South Korea, and was carried out in accordance with the Declaration of Helsinki.

### Subjects

Seven right-handed, non-smoking patients with schizophrenia participated in the study. For the enrollment, patients with schizophrenia were required to have been treated with aripiprazole for at least 6 weeks which are expected for stable therapeutic effects of aripiprazole^12,13^ and to be clinically stable determined with no exacerbations in this period and a total score of <60 in the positive and negative syndrome scale (PANSS)^14^.

After complete description of the study to the subjects, written informed consent was obtained. Screening tests for patients with schizophrenia included physical examinations, vital signs, laboratory tests (hematology, blood chemistry, and urinalysis), and a 12-lead electrocardiogram. A psychiatric interview with the Structured Clinical Interview for DSM-IV-TR Axis I Disorders, Research Version (SCID-I/II) was conducted^15,16^. Subjects with any medically significant abnormality on investigations and/or psychiatric disease were excluded.

### Study design

**Fig. 1:**
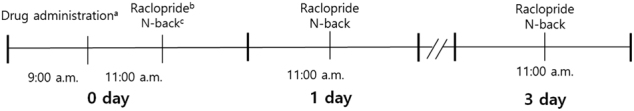
Diagram for the study protocol. **a** Aripiprazole. **b** Positron emission tomomgraphy (PET) scan with [^11^C]raclopride. **c** *N*-back task performed within 30 min of the end of the PET scan

The study followed a prospective study design (Fig. 1). Subjects were required to stay at the Clinical Trial Centre, Seoul National University Hospital and to abstain from caffeine or caffeine-containing products (e.g., coffee, cola, black tea, green tea, and chocolate), grapefruit-containing products, alcohol, and smoking for the duration of study. After fasting for at least 4 h, the subjects received the same oral dose of aripiprazole as they had been regularly prescribed, with 240 ml of water, at 9:00 a.m. Serial PET scans with [^11^C]raclopride were conducted 2, 26, and 74 h after the administration of aripiprazole. Blood samples for the determination of aripiprazole plasma concentration were obtained <5 min before the PET scans. The subjects performed the *N*-back task, a measure of working memory described below, within 30 min of finishing the [^11^C]raclopride PET scans. All the measurements were obtained at the same time point on each occasion, in view of possible diurnal variation in brain activity.

### PET scanning procedure and image analysis

All PET scans were performed on an ECAT EXACT 47 scanner (full-width half-maximum [FWHM] = 4.6 mm) (Siemens-CTI, Knoxville, TN, USA). Before the acquisition of the dynamic scan, a transmission scan was performed using three Ge-68 rod sources for attenuation correction. To measure dopamine receptor occupancy by aripiprazole, dynamic 3D emission scans over 60 min (15 s × 8 frames, 30 s × 16, 60 s × 10, and 240 s × 10) were conducted after a bolus injection of 370–740 mBq [^11^C]raclopride. The data from the dynamic scans were reconstructed in a 128 × 128 × 47 matrix with a pixel size of 2.1 × 2.1 × 3.4 mm by means of a filtered back-projection algorithm, employing a Shepp–Logan filter, with a cutoff frequency of 0.3 cycles/pixel.

Magnetic resonance (MR) images were obtained on a GE Sigma1.5-T scanner. Static PET images, produced by combining all the frames of dynamic images, were co-registered with the MR images of the same individual. The MR images were used to define the regions of interest (ROIs) on the striatum and the reference region (the cerebellum)^17^. The ROIs were drawn on the subject’s T1 MR images by a single rater on ten axial slices for the striatum and cerebellum. We used the transformation parameters obtained by the co-registration of the static PET and MR images with SPM8 and transferred the ROI onto the dynamic PET images to access the time–activity curves for the whole volume of interest (VOI) by applying the parameters.

We calculated D2/3 receptor binding potential (BP_ND_) in the striatum by using a simplified reference tissue model^18,19^. The D2/3 receptor occupancy by aripiprazole was calculated as the percentage reduction of BP_ND_ with drug treatment, compared with the drug-free condition from Equation (1).

1 $${\rm{Occupancy}}\left({\% }\right) = \frac{{{\rm{BP}}_{{\rm{ND}}_{{{{\rm{drug}} - {\rm{free}}}}}} - {{\rm{BP}}}_{{{{\rm{ND}}_{\rm{drug}}}}}}}{{{\rm{BP}}_{{\rm{ND}}_{{{{\rm{drug}} - {\rm{free}}}}}}}} \times 100$$

Because patients with schizophrenia were already taking aripiprazole, we obtained the drug-free BP_ND_ using an inhibitory *E*_max_ model (2) with individual serial BP_ND_ data. We have assessed the reliability of the inhibitory *E*_max_ model to calculate drug-free BP_ND_, finding intraclass correlation coefficients greater than 0.8, which suggests good agreement with measured drug-free BP_ND_^20^.

2 $${\rm{BP}}_{{\rm{ND}}} = {\rm{BP}}_{{{{\rm{ND}}_{{\rm{drug}} - {\rm{free}}}}}} - \frac{{I_{\max } \times {\rm{Conc}}^r}}{{{\rm{IC}}_{50}^r + {\rm{Conc}}^r}}$$

*I*_max_ is the maximum inhibitory effect, Conc is plasma concentration of aripiprazole, IC_50_ is the plasma concentration associated with a 50% decrease of BP_ND_, and *r* is the Hill coefficient. When a very high dose of aripiprazole is administered, BP_ND_ is equal to zero, and it follows from Equation (2) that *I*_max_ is equal to BP_NDdrug-free_. Nonlinear mixed-effects modeling simultaneously estimates fixed effects and random effects in the inhibitory *E*_max_ model. Fixed effects are parameters, including *I*_max_, IC_50_, and Hill coefficient which describe the relationship between the plasma aripiprazole concentration and BP_ND_ in the population. The random effects are composed of inter-individual variability and residual variability.

From the nonlinear mixed-effect modeling, we obtained individual estimates of drug-free BP_ND_ as follows: drug-free BP_ND*i*_ = *I*_max_ × exp(*η*_*i*_ of *I*_max_), where drug-free BP_ND*i*_ indicates the true drug-free BP_ND_ value for the *i*th individual, *I*_max_ is the typical population value of the maximum inhibitory effect, and *η*_*i*_ is inter-individual variability of the maximum inhibitory effect for *i*th individual. The estimation was conducted using NONMEM ver. 7.2.0. software (GloboMax, Ellicott City, MD, USA).

### *N*-back task

The stimuli consisted of numbers (1, 2, 3, and 4) presented in a random sequence and displayed at the points of a diamond-shaped box. In the zero-back task, the non-memory-control condition, subjects had to press a button corresponding to the digit observed at the time. The task had increasing levels of memory load as subjects were required to recollect the stimuli shown as one stimulus (1-back), two stimuli (2-back), or three stimuli (3-back) beforehand while encoding additional incoming stimuli. Each *N*-back task comprised of 20 stimuli and each session comprised four sets of each *N*-back task. The presentation of each *N*-back task in each session was pseudo-randomly assigned. All subjects had a preliminary session to ensure that they understood the task. The subjects were instructed to respond right after the presentation of a number. Performance data were recorded as the number of correct responses and the reaction time for correct responses.

### Statistical analysis

We employed mixed-effects modeling in a repeated-measures analysis to explore the relationship between the D2/3 receptor occupancy by aripiprazole and the performance of the *N*-back task measured in terms of the mean error rate and the reaction time. The D2/3 receptor occupancy, the level of memory load in the *N*-back task (zero-, one-, two-, and three-back), and the interaction between the occupancy and the level of memory load were incorporated into the model as fixed effects, and subjects were modeled as random effects. The performance of the *N*-back task was tested by comparing memory-load conditions (one-, two-, and three-back) with individual non-memory-control conditions (zero-back) measured at each time point to exclude the influence of aripiprazole on motor performance.

## Results

A total of seven patients, with five being women, were enrolled in the study. Mean (±SD) age, height, and body weight of patients was 32.0 ± 10.5 years, 163.3 ± 11.1 cm, and 62.8 ± 17.5 kg, respectively. The mean (±SD) maintenance dose of aripiprazole for patients was 14.2 ± 12.0 mg (from 2 mg to 30 mg) and the mean corresponding period for the maintenance dose was 828.6 ± 1427.0 weeks (from 10 to 3744 weeks). The mean PANSS total score ( ± SD) was 43.7 ± 8.8 (Table 1). Patients had no concomitant medication.

**Table 1:** Demographic data and clinical characteristics

	Subjects (*n* = 7)
Demographic characteristics	
Age (years)	32(10.5)
Female gender, *n* (%)	5(71.4)
Height (cm)	163.3(11.1)
Weight (kg)	62.8(17.5)
Clinical characteristics	
Aripiprazole dosage (mg)	14.2(12.0)
Duration of illness (weeks)	828.6(1427.0)
PANSS total	43.7(8.8)
Positive	8.1(1.1)
Negative	13.3(5.0)
General	22.4(3.7)

All variables are presented as means (±SD), or *n* (%).

**Fig. 2:**
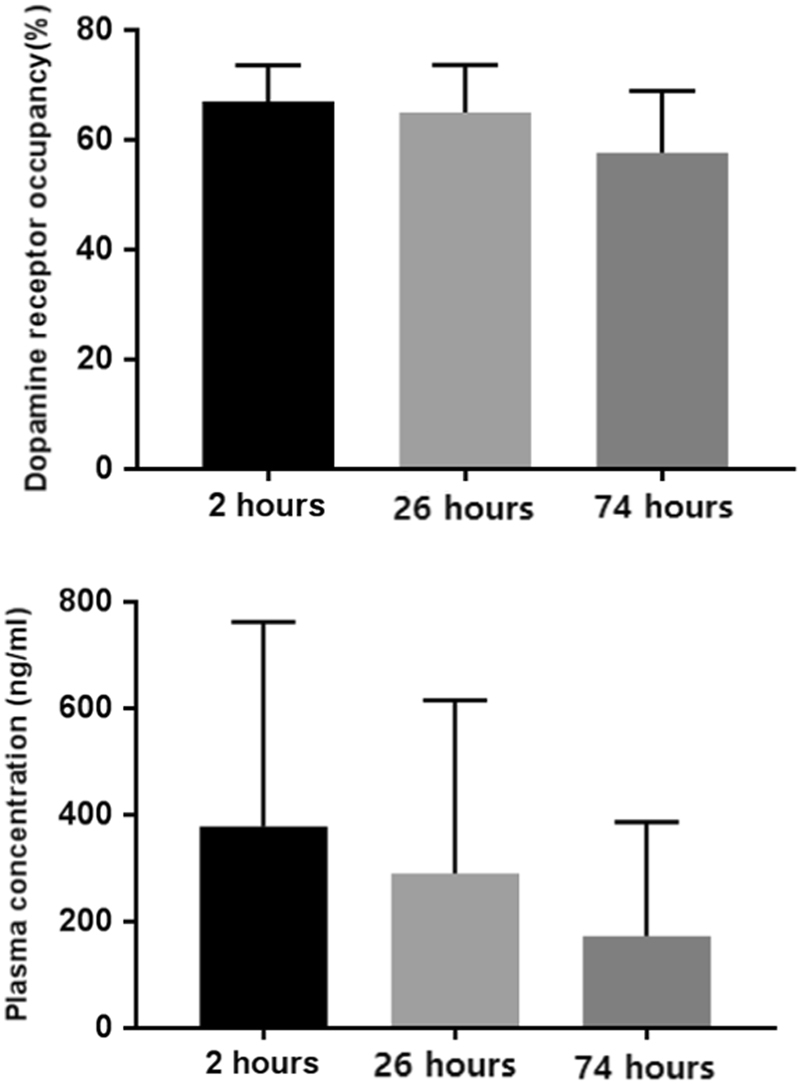
Dopamine D2/3 receptor occupancy and average plasma concentrations of aripiprazole according to time. The error bar indicates standard deviation

The average plasma concentrations (±SD) of aripiprazole were 378.3 ± 383.1 ng/ml, 289.9 ± 325.2 ng/ml, and 172.5 ± 213.7 ng/ml at 2 h, 26 h, and 74 h after drug administration, respectively. The mean D2/3 receptor occupancies (±SD) by aripiprazole were 66.9 ± 6.7%, 65.0 ± 8.6%, and 57.7 ± 11.2% at 2 h, 26 h, and 74 h after drug administration, respectively (Fig. 2).

**Fig. 3:**
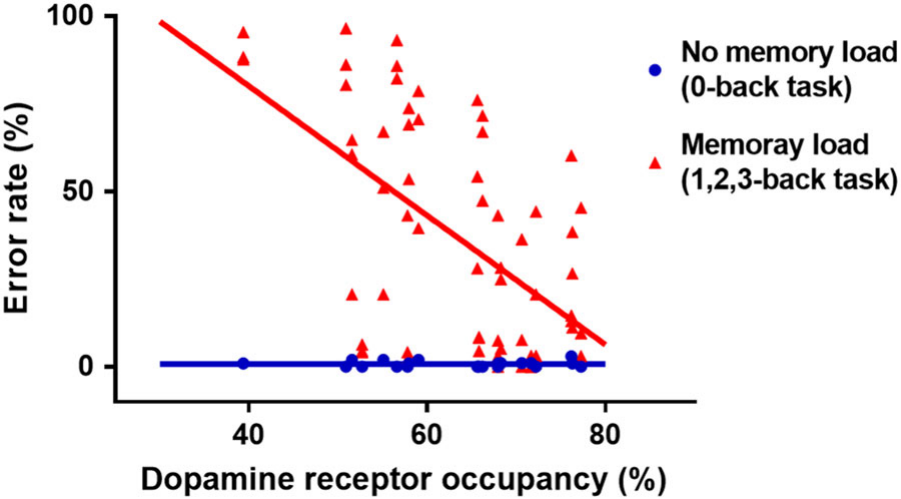
The relationship between dopamine D2/3 receptor occupancy and mean error rates in the no-memory load and memory-load conditions of the *N*-back task after aripiprazole administration. This shows an inverse relationship between dopamine D2/3 receptor occupancy and error rate, indicating that better performance is associated with higher D2/3 receptor blockade (memory load, *ß* = −2.236,* t* = −6.631, df = 53.947, and *p* = 0.001)

**Fig. 4:**
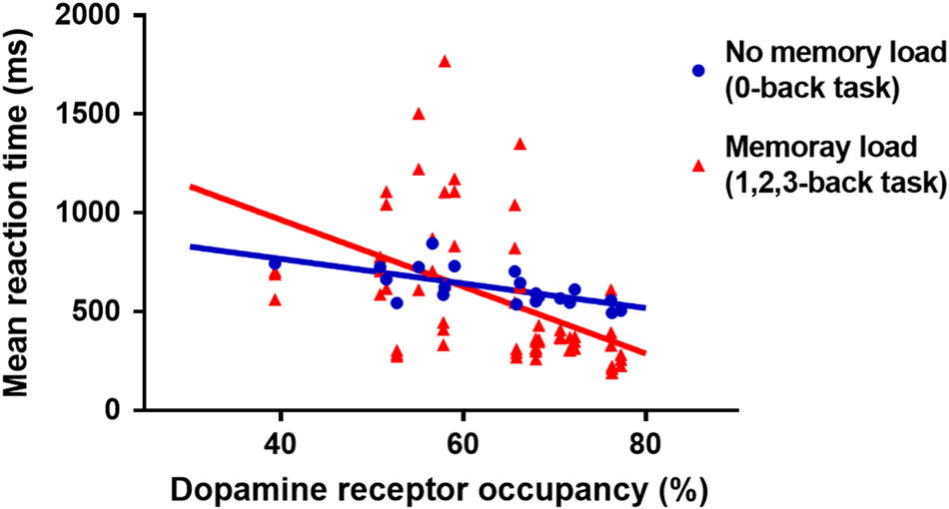
The relationship between dopamine D2/3 receptor occupancy and mean reaction times for correct responses in the no-memory load and memory-load conditions of the *N*-back task after aripiprazole administration. This shows an inverse relationship between D2/3 receptor occupancy and reaction time, indicating that better performance is associated with higher D2/3 receptor blockade (memory load, *ß* = −9.567, *t* = −2.808, df = 29.967, and *p* = 0.009)

Table 2 and 3 show the mean error rates and the mean reaction times after the administration of aripiprazole (Supplementary Table 1). There were no differences according to the observation time points in the mean error rates (0-back: *F* = 0.76, df = 2, and *p* = 0.481; 1-back: *F* = 0.01, df = 2, and *p* = 0.989; 2-back: *F* = 0.04, df = 2, and *p* = 0.962; and 3-back: *F* = 0.45, df = 2, and *p* = 0.646) and the mean reaction time (0-back: *F* = 0.68, df = 2, and *p* = 0.521; 1-back: *F* = 0.69, df = 2, and *p* = 0.514; 2-back: *F* = 0.03, df = 2, and *p* = 0.966; and 3-back: *F* = 0.04, df = 2, and *p* = 0.959). Compared with the performance in the zero-back task, the performance in memory-load conditions (one-, two, three-back task) was significantly related to D2/3 receptor occupancy by aripiprazole (error rate: *ß* = −2.236, *t* = −6.631, df = 53.947, and *p* = 0.001; reaction time: *ß* = –9.567, *t* = –2.808, df = 29.967, and *p* = 0.009) (Figs. 3 and 4). A post hoc analysis found that error rates were significantly correlated with all memory-loaded tasks (1-back, *ß* = –2.131, *t* = −4.137, df = 18.341, and *p* = 0.001; 2-back, *ß* = –3.111, *t* = −5.943, df = 13.454, and *p* = 0.001; and 3-back, *ß* = −1.512, *t* = −2.332, df = 17.878, and *p* = 0.032) (Supplementary Figure 1), while reaction times were significantly related with the D2/3 receptor occupancy in the two-back condition (1-back, *ß* = −5.752, *t* = −1.560, df = 16.938, and *p* = 0.137; 2-back, *ß* = −21.453, *t* = −3.153, df = 16.623, and *p* = 0.006; and 3-back, *ß* = −13.311, *t* = −1.533, df = 18.347, and *p* = 0.142) (Supplementary Figure 2).

**Table 2:** The mean error rates (±SD) of the *N*-back task

	Hours		
	2	26	74
0-back, mean (SD), %	1.0(1.2)	0.4(0.8)	0.8(0.7)
1-back, mean (SD), %	24.7(29.2)	23.7(29.7)	26.1(32.6)
2-back, mean (SD), %	40.1(30.4)	35.1(36.5)	36.5(35.8)
3-back, mean (SD), %	59.1(24.9)	44.6(34.3)	45.8(35.3)

All variables are presented as mean (±SD).

**Table 3:** The mean reaction times (±SD) for correct responses

	Hours		
	2	26	74
0-back, mean (SD), ms	656.5(108.0)	611.4(90.0)	601.4(83.8)
1-back, mean (SD), ms	595.5(306.9)	472.4(195.7)	461.4(190.6)
2-back, mean (SD), ms	617.0(311.3)	563.9(456.0)	578.8(391.3)
3-back, mean (SD), ms	591.9(289.6)	614.5(474.6)	661.3(562.9)

All variables are presented as mean (±SD).

## Discussion

To our knowledge, this is the first report of the relationship between the D2/3 receptor occupancy by aripiprazole and working-memory performance in patients with schizophrenia. Our main findings are that greater striatal D2/3 receptor occupancy by aripiprazole was related with lower error rates and shorter reaction time in the *N*-back task. These results indicate that aripiprazole’s occupancy of striatal D2/3 receptors is related to improvements in cognitive function, and suggest that it has potential to treat cognitive dysfunction in patients with schizophrenia.

Indeed, previous studies have reported a positive effect of aripiprazole on brain or cognitive function in patients with schizophrenia^4,21^. After switching from first-generation antipsychotics (FGA) such as haloperidol to aripiprazole, the blood-oxygen-level-dependent signal measured in the anterior cingulate cortex in patients with schizophrenia increased during cognitive function tasks^21^. They also showed performance improvement in memory, attention, and executive function tests after switching to aripiprazole^22,23^. Moreover, verbal cognitive function in patients improved after switching from second-generation antipsychotics and other FGAs to aripiprazole^3,24^.

However, it has been a challenge to disentangle the specific effects of aripiprazole on patients’ cognitive function from secondary ones due to the improvement of other symptoms and its less-sedative effects following the switch from other antipsychotics to aripiprazole in previous studies^3,21,24^. We recruited patients with schizophrenia who were clinically stable with the treatment with aripiprazole, and all patients were required to stay at the Clinical Trial Center for the study period. This guaranteed that the cognitive measurements were obtained in clinically similar conditions across the observations.

We found that greater dopamine receptor occupancy by aripiprazole was related with better working-memory performance in terms of error rate and reaction time. This extends previous findings that striatal dopamine receptor occupancy is related to improvement in positive symptoms in schizophrenia^25,26^. Striatal hyperdopaminergia is a well-replicated finding in schizophrenia^11,27^. Preclinical models show that increased striatal dopaminergic transmission through overexpression of striatal D2 receptors is associated with impairments in working memory^28^. Thus, excess striatal dopaminergic function could contribute to working-memory impairments in schizophrenia, and aripiprazole could ameliorate this by reducing excess striatal dopaminergic neurotransmission. This is supported by a meta-analysis that atypical antipsychotic drugs enhanced cognitive function in schizophrenia^29^, though recent studies demonstrated that high D2/3 receptor occupancy by dopamine antagonists, including risperidone, olanzapine, and ziprasidone might be related with cognitive impairment in schizophrenia^30–33^, and that reduction of antipsychotic drugs improved cognitive dysfunction^30^.

We measured D2/3 receptor occupancy by aripiprazole in the striatum. The extrastriatal occupancies by aripiprazole measured by using [^18^F]fallypride PET were reported to be well correlated with the striatal ones by [^11^C]raclopride PET, though the extrastriatal occupancies were higher than striatal ones^34^. It enables us to speculate similar trends of prefrontal occupancies with striatal ones. The mesocortical dopamine system, providing a widespread innervation to the dorsolateral prefrontal cortex, is thought to be defective in patients with schizophrenia, leading to chronic deficits in prefrontal dopamine function^35^. Furthermore, the depletion is believed to be related with cognitive impairment in patients with schizophrenia^36,37^. Thus, aripiprazole, a dopamine-partial agonist, could act as an agonist in the prefrontal hypodopaminergic state and may improve cognitive dysfunction in patients with schizophrenia, as shown in the previous report that the partial D2 agonism in aripiprazole enhanced the activation of the dorsolateral prefrontal cortex associated with trends for improved discriminability and speeded reaction times in the working-memory task^38^. The effect of aripiprazole on motor function could have confounded the result of *N*-back tasks^39^. However, the performance in the zero-back task (no memory load) was not affected by the receptor occupancy by aripiprazole (Figs. 3 and 4). Thus, this is unlikely to be the case.

Aripiprazole also has appreciable affinities for 5-HT1A and 5-HT2A receptors and acts as a partial 5-HT1A agonist and 5-HT2A antagonist^10^. There are several reports regarding the relationship between the 5-HT system and cognitive function. For example, it was reported that the partial 5-HT1A agonist decreased verbal recall^40^ but without affecting abilities in memory, executive planning, impulse control, decision making, or cognitive flexibility^41^. In addition, while the 5-HT1A receptor agonist yielded no effect on executive function^42^, the 5-HT2A receptor antagonist impaired spatial working memory of the subject under evaluation^40^. We did not measure aripiprazole’s pharmacological effect on the 5-HT system. Thus, it is required to investigate into aripiprazole’s potential role on the 5-HT system and its relationship with the cognitive function, though the 5-HT1A and 5-HT2 receptor occupancies by aripiprazole were reported to be even lower than D2/3 occupancy and to exhibit no relationship with dosage and plasma concentration of aripiprazole^43,44^.

When interpreting the results, some limitations should be taken into consideration. First, we investigated the relationship between the receptor occupancy and working memory in a relatively small sample. However, we repeatedly measured the receptor occupancy and working memory in each patient. This controls individual variation of cognitive function, consequently increasing the statistical power. Second, we observed the relationship for 3 days after the last administration of aripiprazole in patients with schizophrenia. Though the receptor occupancies obtained at 74 h after aripiprazole administration were still high, it is necessary to consider the possibility of withdrawal symptoms affecting cognitive function after discontinuation^45^. Case studies reported that patients experienced symptoms of sudden-onset lightheadedness, intermittent nausea, insomnia, irritability, generalized muscular twitches, intense anxiety, and dysphoria from the cessation of aripiprazole^46,47^. Only one study reported that the cognitive function of patients diagnosed with schizophrenia deteriorated after 3 or 4 weeks following the discontinuation of antipsychotic drugs, including risperidone, clozapine, olanzapine, or quetiapine^45^. This result cannot be directly compared with the current one in that the antipsychotic drugs of interest were dopamine antagonists, and that the duration of antipsychotic discontinuation was longer than that of the current study which allowed washout of antipsychotic drugs, leading to symptomatic aggravations^45^. However, the influence of withdrawal symptoms on cognitive function could not be excluded completely, and further investigation is required. Finally, the practice effect could have influenced the outcome of repeated *N*-back tasks. However, a recent meta-analysis reported that the magnitude of the practice effect on the measurement was minimal in patients with schizophrenia^48^. In addition, the error rate and the response time were not different across the observation time points, which indicated that the practice effect was unlikely to influence the result.

Nevertheless, this study is noteworthy in suggesting aripiprazole’s direct effects on cognitive function in patients with schizophrenia by overcoming limitations in many studies, such as individual variation of performance in subjects and drug-occupancy levels in their brains, as well as secondary effects from the improvement of psychotic symptoms.

In summary, the greater dopamine receptor occupancy by aripiprazole was related with better performance in the working-memory task, suggesting that aripiprazole can enhance cognitive function in patients with schizophrenia whose dopaminergic function is defective.

## Electronic supplementary material

Supplementary Table 1

Supplementary Figure 1

Supplementary Figure 2
