# Supplementary material for: The relationship between dopamine receptor blockade and cognitive performance in schizophrenia: a [11C]-raclopride PET study with aripiprazole
Source: Transl Psychiatry. 2018 Apr 24;8:87. doi: 10.1038/s41398-018-0134-6 (PMC5913226; doi:10.1038/s41398-018-0134-6)
Supplement: Supplementary file 1 — Supplementary Table 1 [file 41398_2018_134_MOESM1_ESM.docx]

Supplementary Table 1. Individual mean error rate, mean reaction time and dopamine D2/3 receptor occupancy

|  |  | Mean error rates (%) | | | Mean reaction times (msec) | | | Dopamine receptor occupancy (%) | | |
| --- | --- | --- | --- | --- | --- | --- | --- | --- | --- | --- |
| Pt | N-back task | 2  hours | 26 hours | 72 hours | 2  hours | 26 hours | 72 hours | 2  hours | 26 hours | 72 hours |
| 1 | 0 | .0 | .0 | 1.0 | 845.8 | 725.8 | 743.9 | 56.6 | 50.9 | 39.3 |
|  | 1 | 82.3 | 80.4 | 87.6 | 872.6 | 778.2 | 704.7 | 56.6 | 50.9 | 39.3 |
|  | 2 | 85.9 | 86.2 | 88.3 | 870.3 | 705.1 | 690.5 | 56.6 | 50.9 | 39.3 |
|  | 3 | 93.2 | 96.6 | 95.5 | 706.0 | 587.5 | 561.9 | 56.6 | 50.9 | 39.3 |
| 2 | 0 | .0 | .0 | 1.0 | 704.9 | 645.3 | 624.4 | 65.6 | 66.2 | 58.0 |
|  | 1 | 28.1 | 47.4 | 53.6 | 1041.5 | 623.4 | 636.9 | 65.6 | 66.2 | 58.0 |
|  | 2 | 54.3 | 67.0 | 69.1 | 822.3 | 642.6 | 1106.4 | 65.6 | 66.2 | 58.0 |
|  | 3 | 76.1 | 71.6 | 73.9 | 547.9 | 1350.4 | 1769.1 | 65.6 | 66.2 | 58.0 |
| 3 | 0 | 3.0 | .0 | 1.0 | 556.9 | 505.5 | 494.8 | 76.2 | 77.3 | 76.3 |
|  | 1 | 14.6 | 3.1 | 11.2 | 394.9 | 283.3 | 223.9 | 76.2 | 77.3 | 76.3 |
|  | 2 | 13.0 | 9.6 | 26.6 | 329.1 | 256.7 | 213.0 | 76.2 | 77.3 | 76.3 |
|  | 3 | 60.2 | 45.5 | 38.5 | 610.1 | 225.5 | 190.6 | 76.2 | 77.3 | 76.3 |
| 4 | 0 | 1.0 | .0 | .0 | 576.1 | 538.2 | 543.6 | 68.3 | 65.8 | 52.7 |
|  | 1 | 5.2 | 8.2 | 4.1 | 348.1 | 291.7 | 273.9 | 68.3 | 65.8 | 52.7 |
|  | 2 | 28.3 | 8.5 | 6.4 | 430.5 | 268.4 | 286.5 | 68.3 | 65.8 | 52.7 |
|  | 3 | 25.0 | 4.5 | 4.5 | 358.8 | 311.5 | 304.9 | 68.3 | 65.8 | 52.7 |
| 5 | 0 | 1.0 | 1.0 | 1.0 | 567.8 | 546.5 | 552.7 | 70.6 | 71.7 | 67.9 |
|  | 1 | .0 | 3.1 | 1.0 | 366.3 | 369.0 | 362.9 | 70.6 | 71.7 | 67.9 |
|  | 2 | 7.6 | .0 | 2.1 | 407.5 | 309.7 | 315.6 | 70.6 | 71.7 | 67.9 |
|  | 3 | 36.4 | .0 | .0 | 372.2 | 303.9 | 312.3 | 70.6 | 71.7 | 67.9 |
| 6 | 0 | 2.0 | 2.0 | 2.0 | 731.5 | 726.2 | 664.5 | 59.0 | 55.1 | 51.6 |
|  | 1 | 39.6 | 20.6 | 20.6 | 832.0 | 611.6 | 616.8 | 59.0 | 55.1 | 51.6 |
|  | 2 | 70.7 | 67.0 | 60.6 | 1109.3 | 1503.9 | 1106.9 | 59.0 | 55.1 | 51.6 |
|  | 3 | 78.7 | 51.1 | 64.8 | 1173.3 | 1223.2 | 1044.7 | 59.0 | 55.1 | 51.6 |
| 7 | 0 | .0 | .0 | .0 | 612.4 | 592.2 | 585.7 | 72.2 | 68.0 | 57.8 |
|  | 1 | 3.1 | 3.1 | 4.1 | 313.4 | 349.7 | 410.8 | 72.2 | 68.0 | 57.8 |
|  | 2 | 20.7 | 7.4 | 2.1 | 349.9 | 261.2 | 332.8 | 72.2 | 68.0 | 57.8 |
|  | 3 | 44.3 | 43.2 | 43.2 | 374.7 | 299.6 | 445.6 | 72.2 | 68.0 | 57.8 |

Abbreviation: Pt., Patient
