# Supplementary material for: The relationship between dopamine receptor blockade and cognitive performance in schizophrenia: a [11C]-raclopride PET study with aripiprazole
Source: Transl Psychiatry. 2018 Apr 24;8:87. doi: 10.1038/s41398-018-0134-6 (PMC5913226; doi:10.1038/s41398-018-0134-6)

Supplementary Figure 1. The relationship between dopamine D2/3 receptor occupancy and the mean error rates for each level of the N-back task after aripiprazole administration. This shows that each memory load level is inversely related to D2/3 receptor occupancy (1-back, ß=-2.131, t=-4.137, df=18.341, p=0.001; 2-back, ß=-3.111, t=-5.943, df=13.454, p=0.001; 3-back, ß=-1.512, t=-2.332, df=17.878, p=0.032).


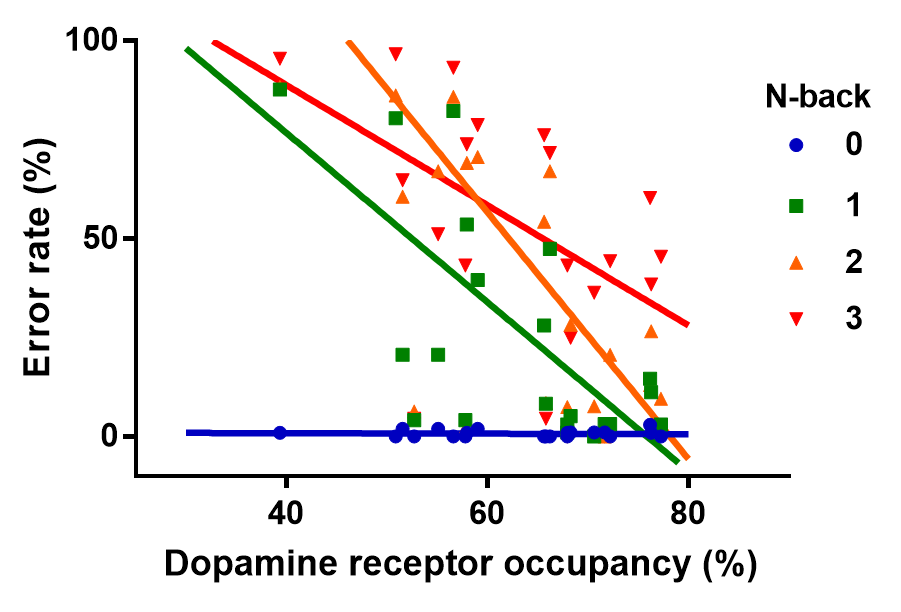

Supplement: Supplementary file 2 — Supplementary Figure 1 [file 41398_2018_134_MOESM2_ESM.docx]
