# Supplementary material for: The relationship between dopamine receptor blockade and cognitive performance in schizophrenia: a [11C]-raclopride PET study with aripiprazole
Source: Transl Psychiatry. 2018 Apr 24;8:87. doi: 10.1038/s41398-018-0134-6 (PMC5913226; doi:10.1038/s41398-018-0134-6)
Supplement: Supplementary file 3 — Supplementary Figure 2 [file 41398_2018_134_MOESM3_ESM.docx]

Supplementary Figure 2. The relationship between dopamine D2/3 receptor occupancy and the mean reaction time for correct responses in each level of the N-back task after aripiprazole administration. This shows a significant inverse relationship between reaction time and D2/3 receptor occupancy for the 2-back level, but not the 1-back or 3-back conditions (1-back, ß=-5.752, t=-1.560, df=16.938, p=0.137; 2-back, ß=-21.453, t=-3.153, df=16.623, p=0.006; 3-back, ß=-13.311, t=-1.533, df=18.347, p=0.142).

| 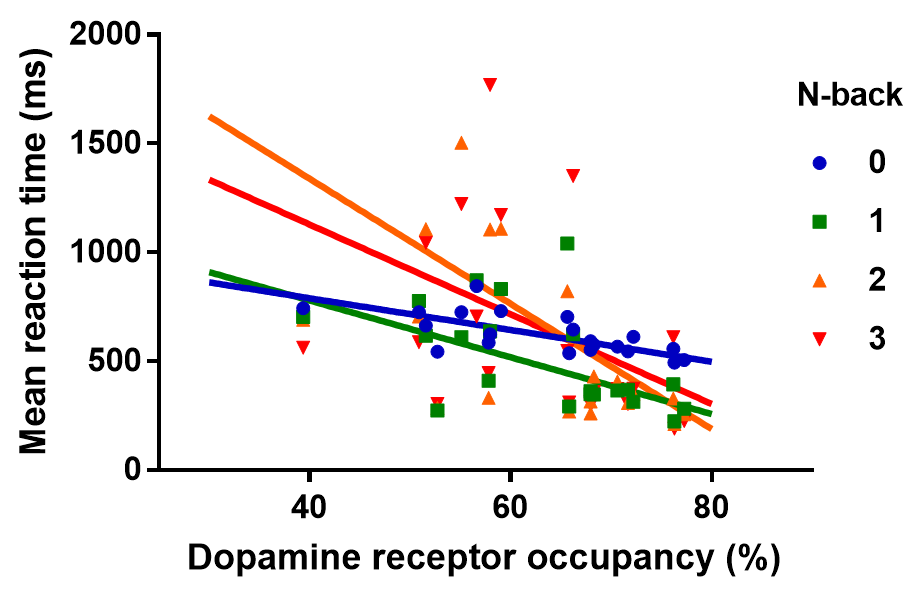 |
| --- |
